# Finite element analysis of a novel anatomical locking plate for scapular neck fracture

**DOI:** 10.1186/s13018-023-03743-3

**Published:** 2023-03-31

**Authors:** Feifan Xiang, Yukun Xiao, Daiqing Wei, Xiaoqi Tan, Siyuan He, Liang Luo, Yunkang Yang

**Affiliations:** 1 Department of Orthopedics, The Affiliated Hospital of Southwest Medical University, Luzhou, 646000 China; 2 Sichuan Provincial Laboratory of Orthopaedic Engineering, Luzhou, 646000 China; 3 State Key Laboratory of Quality Research in Chinese Medicine, Macau University of Science and Technology, Macau, 999078 China; 4 Department of Dermatology, The Affiliated Hospital of Southwest Medical University, Luzhou, 646000 China; 5 Department of Ophthalmology, The Second Affiliated Hospital of Chongqing Medical University, Chongqing, 400010 China; 6 Department of Joint Surgery, People’s Hospital of Chongqing Liang Jiang New Area, Chongqing, 401121 China

**Keywords:** Scapular neck fractures, Anatomical locking plate, Reconstruction plate, Biomechanical characteristics, Finite element analysis

## Abstract

**Objectives:**

Reconstruction plates (RPs) are commonly used in scapular neck fractures (SNFs): however, RPs have many defects. In this study, we evaluated a newly designed scapular neck anatomical locking compression plate (SNALCP).

**Methods:**

An SNF finite element model (Miller-type IIB) was constructed. Plates were subsequently implanted into the scapula and fixed with screws that were grouped according to the plate used: SNALCP (A) and RP (B). Finally, loads were applied to record and analyze performance.

**Results:**

Under lateral, anteroposterior, and vertical compression loads, the maximum von Mises stresses on the scapula and implants of group A were smaller than those of group B. There were some differences in stress distribution between the two groups.

**Conclusions:**

SNALCP can effectively reduce the stress of the scapula and implant, making stress distribution more uniform and continuous, and has mechanical conduction advantages. Compared to RP, it provides improved stability and more reliable fixation.

**Supplementary Information:**

The online version contains supplementary material available at 10.1186/s13018-023-03743-3.

## Introduction

Scapular neck fractures (SNFs) account for approximately 7%–25% of all scapular fractures, and 1% of all general fractures. SNFs are predominantly caused by high-energy violent injuries, such as traffic accidents, falling from high places, and trauma from heavy objects, or the indirect forces of the impact of the humeral head on the scapular glenoid when falling on the side [1–3]. In recent years, with the increasing incidence of high-energy trauma, SNFs have become more common in patients with multiple trauma [4].

The Miller classification of SNFs based on the location of the fracture line is most commonly used in clinics [5]. Type IIB SNF is a more common type of fracture in clinical practice. Most SNFs can be treated conservatively by suspension or abductive traction; however, many studies have reported a higher risk of poor prognosis, especially in the presence of significant fracture displacement. As such, surgical intervention should be considered for severely displaced SNFs [3, 4, 6, 7].

Currently, many surgical implant schemes can be used to treat SNFs, including reconstruction plates (RPs), straight locking plates, distal radius T-plates, and Y-locking plates [4, 8–10]. RPs are the most commonly used as they provide relatively reliable internal fixation and general functional results [3, 9, 11]. However, RP does not conform to the anatomic shape of the scapular neck, and the strength of RP is also affected by the need for repeated adjustments. The scapular neck is a cancellous bone with a deep location; in most cases, only one or two screws can be placed in the scapular neck with RP. The direction of the screws is difficult to control, and they can easily penetrate the joint cavity. This can easily lead to a lack of internal fixation control, which affects the stability of the fractured end, especially for scapular neck comminuted or osteoporotic fractures [12, 13].

**Fig. 1:**
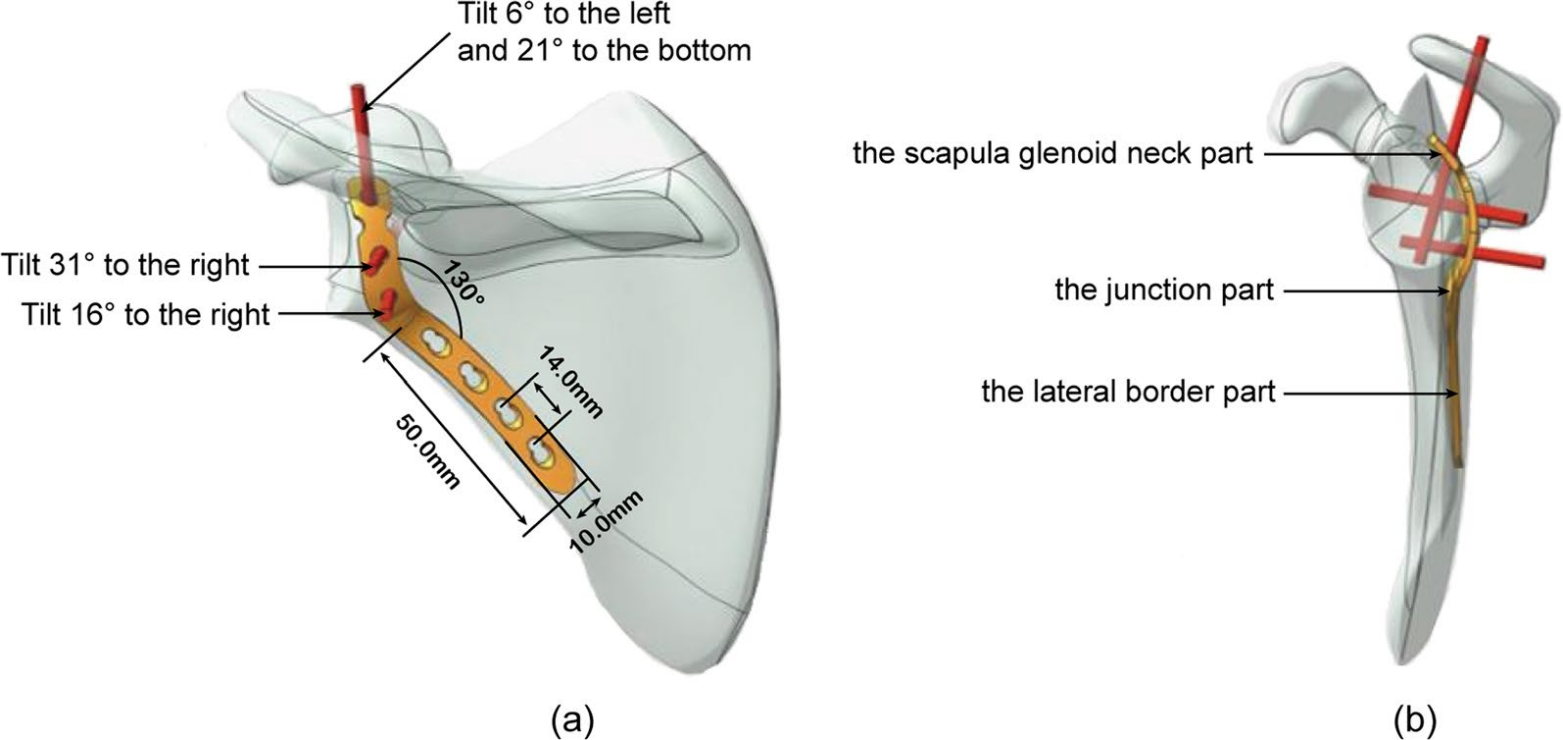
Diagram showing the design and main parameters of the new scapular neck anatomical locking plate. **a** Frontal view. **b** Side view

In a previous study, we developed an understanding of the anatomical characteristics of the scapular neck and the important parameters of the surrounding tissues through preliminary basic research on the scapula [14]. We designed a new scapular neck anatomical locking compression plate (SNALCP, Fig. 1) to solve the above problems, for which we obtained a national utility model invention patent (Patent Number: 20221488758.0). SNALCP conforms to the anatomical characteristics of the scapular neck. The plate runs from the scapular neck along the lateral border, and is divided into three parts: the scapular glenoid neck, junction, and lateral border. The scapular glenoid neck part is arched and perfectly fits with the arc of the scapular glenoid neck. A locking nail is designed above the center of the glenoid at the top of the plate, and the screw hole direction is tilted 6° to the left and 21° to the bottom. Underneath, it is designed with two locking screws in the direction of the coracoid process. The second screw is located in the center of the glenoid neck, and is angled 31° to the right. The third screw is located near the junction part and is angled 16° to the right. The three staggered locking screws realize the three-dimensional fixation of scapular glenoid neck. For the junction part, the angle is designed to match the abduction angle of the scapula (the frontal view is consistent with the abduction angle of 130°). The overall clockwise torsion from the scapular glenoid neck part to the junction part is 5°. The lateral border part is consistent with the back of the lateral border of the scapula. Four common locking joint holes have been designed, and the locking method can be selected according to the patient’s needs.

We hypothesized that SNALCP would have better biomechanical properties compared to RP. Therefore, this study aimed to use finite element analysis to compare the biomechanical characteristics of SNALCP and RP in treating SNFs to lay a foundation for future clinical trials.

## Materials and methods

### Finite element analysis: collection of imaging data

Informed consent was obtained from all participants of this study, and the study was approved by the Medical Ethics Committee of the Affiliated Hospital of Southwest Medical University (Approval Number: KY2022067). A healthy adult male volunteer aged 40 years weighing 75 kg and with a height of 175 cm was chosen as the model. The scapula was scanned using spiral computed tomography (CT), and the thickness of the scanning layer was 1 mm. The data were saved in the Digital Imaging and Communications in Medicine (DICOM) format.

### Finite element models and implants

**Fig. 2:**
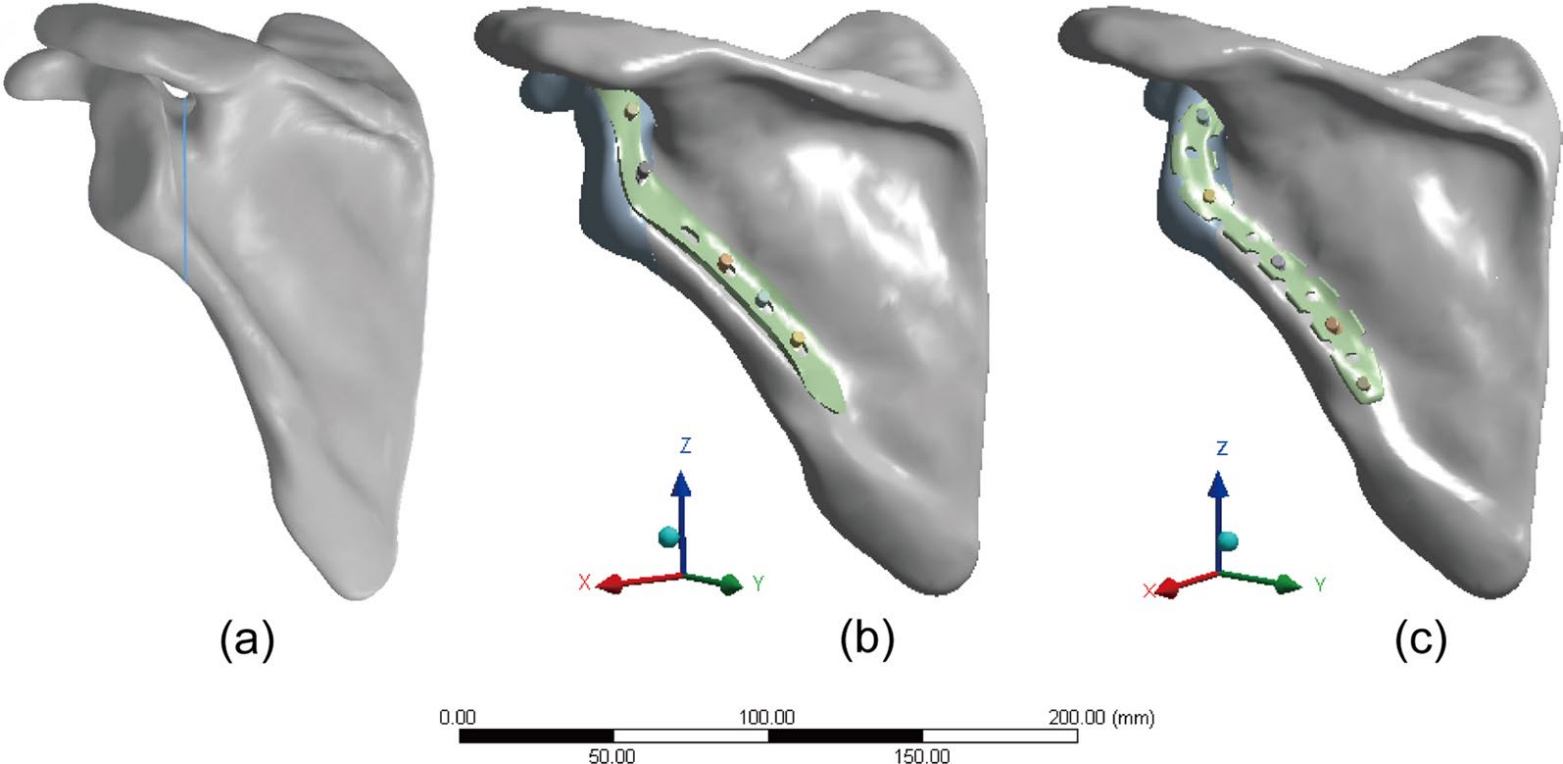
**a** Scapular neck fracture models. **b** Group A (SNALCP). **c** Group B (RP)

Mimics 17.0 software (Materialise, Belgium) was applied to reconstruct a three-dimensional (3D) model of the scapula from the CT images. The model was subsequently imported into Geomagic2017 software (Geomagic, USA), and subdivided into triangular facets, noise reduction, and smooth processing. Thereafter, this model was imported into the Solidworks2017 (Dassault System, France) to simulate the type IIB SNF model [11] (Fig. 2a).

Next, two types of implants were constructed using Solidworks2017 software and divided into group A (SNALCP, Fig. 2b) and group B (RP, Fig. 2c). Standard surgical techniques were simulated to place the two types of implants into the SNF models. The parameters of the SNALCP (7-holes) are shown in Fig. 1, while the RP (10-holes) measured 124 mm × 10 mm (Waston Medical Instrument Co., Ltd., China; Additional file 1).

### Volume mesh generation

Finite-element models were constructed using linear tetrahedrons. We set the number of nodes and elements of the two models based on the 3D finite element models of Groups A and B (Table 1).

**Table 1:** Number of nodes and elements in the meshes of the two groups of models

	Group A (SNALCP)	Group B (RP)
Number of nodes	201,297	160,209
Number of elements	118,510	93,512

### Assignment of material properties

In this experiment, all materials (including the bone, plates, and screws) were assumed to be linearly elastic, isotropic, and homogeneous. The specific parameters, which were decided in accordance with the existing literature [15] are listed in Table 2.

**Table 2:** Material properties of the components in this study

Component	Young’s modulus (MPa)	Poisson’s ratio
Scapula	9000	0.3
Steel plate	110,000	0.3
Screw	110,000	0.3

### Boundary and loading conditions

The 3D models constructed as described above were analyzed using the finite element analysis software ANSYS Workbench 17.0. A coefficient of friction of 0.37 was assigned between the bone and plate contacts, and a value of 1.0 was assigned between the broken bone fragments (i.e., at fracture interfaces) of the bone and plate contacts. The contact behaviors of the screw and plate interfaces and screw and bone interfaces were defined as ties [16]. All contact elements were defined as deformable elements.

**Fig. 3:**
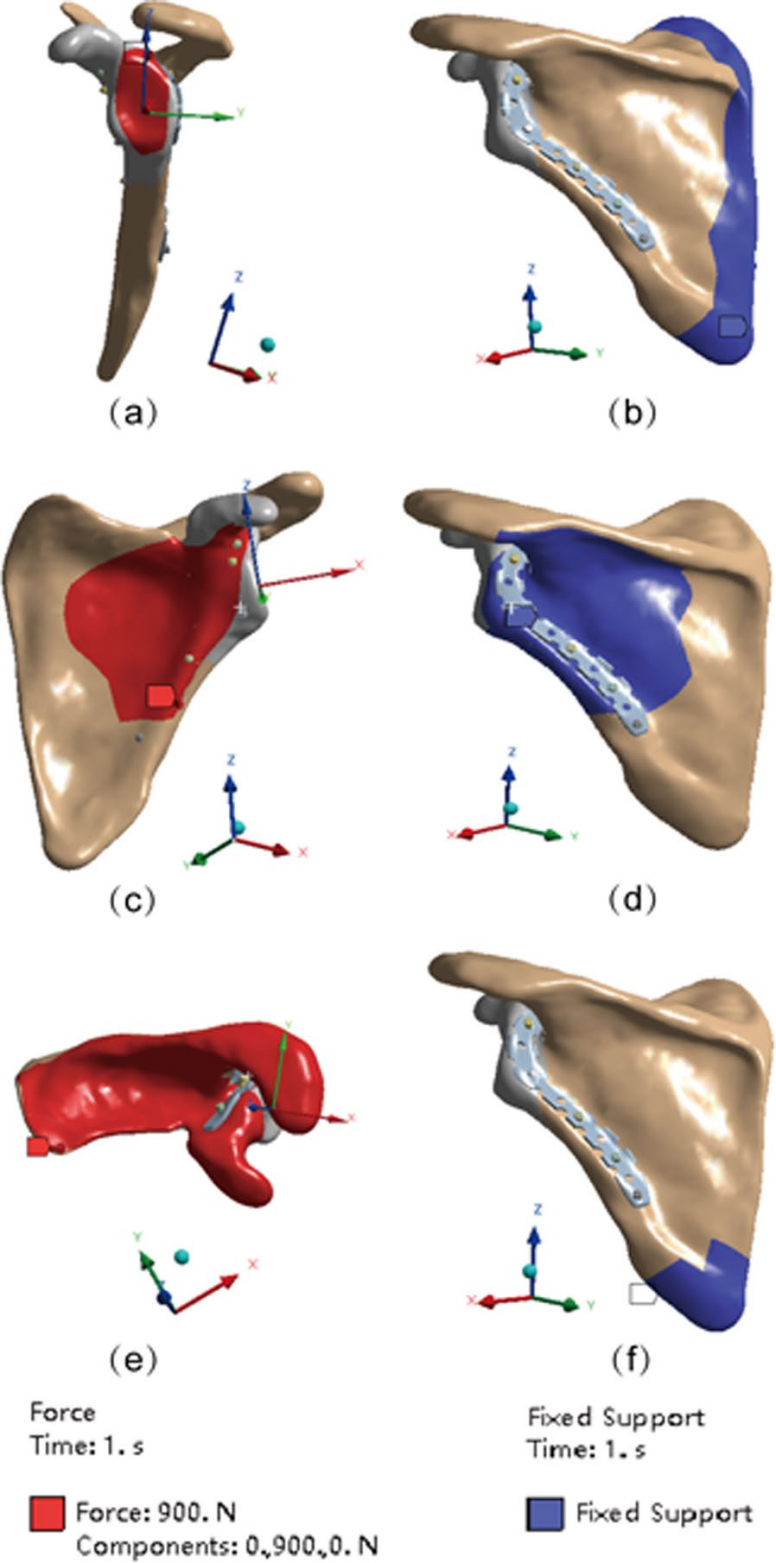
Loading and boundary condition of the 3D model. Red represents the force surface and blue represents boundary condition. **a**, **b** Force surface and boundary condition for Lateral compression load. **c**, **d** Force surface and boundary condition for anteroposterior compression load. **e**, **f** Force surface and boundary condition for vertical compression load

Three loads in different directions were applied to the two sets of models and the corresponding boundary conditions were set (Fig. 3). To simulate the most common injury mechanism of the scapular neck (a fall landing on one side with trauma transmitted through the proximal humerus to the scapular neck), a 900 N lateral compression load was applied along the axis of the scapula with the scapula glenoid neck as the force surface. The medial border of the scapula was fixed (Fig. 3a, b). To simulate direct trauma from behind and above, a 900 N anteroposterior compression load (Fig. 3c, d) and a vertical compression load (Fig. 3e, f) were set (Additional file 1).

### Observation index

The maximum von Mises stresses and stress distribution on the scapula and implants, as well as the maximum displacements of the fracture gaps, were recorded and analyzed by applying loads in different directions to the two models.

## Results

### Scapula stress

**Fig. 4:**
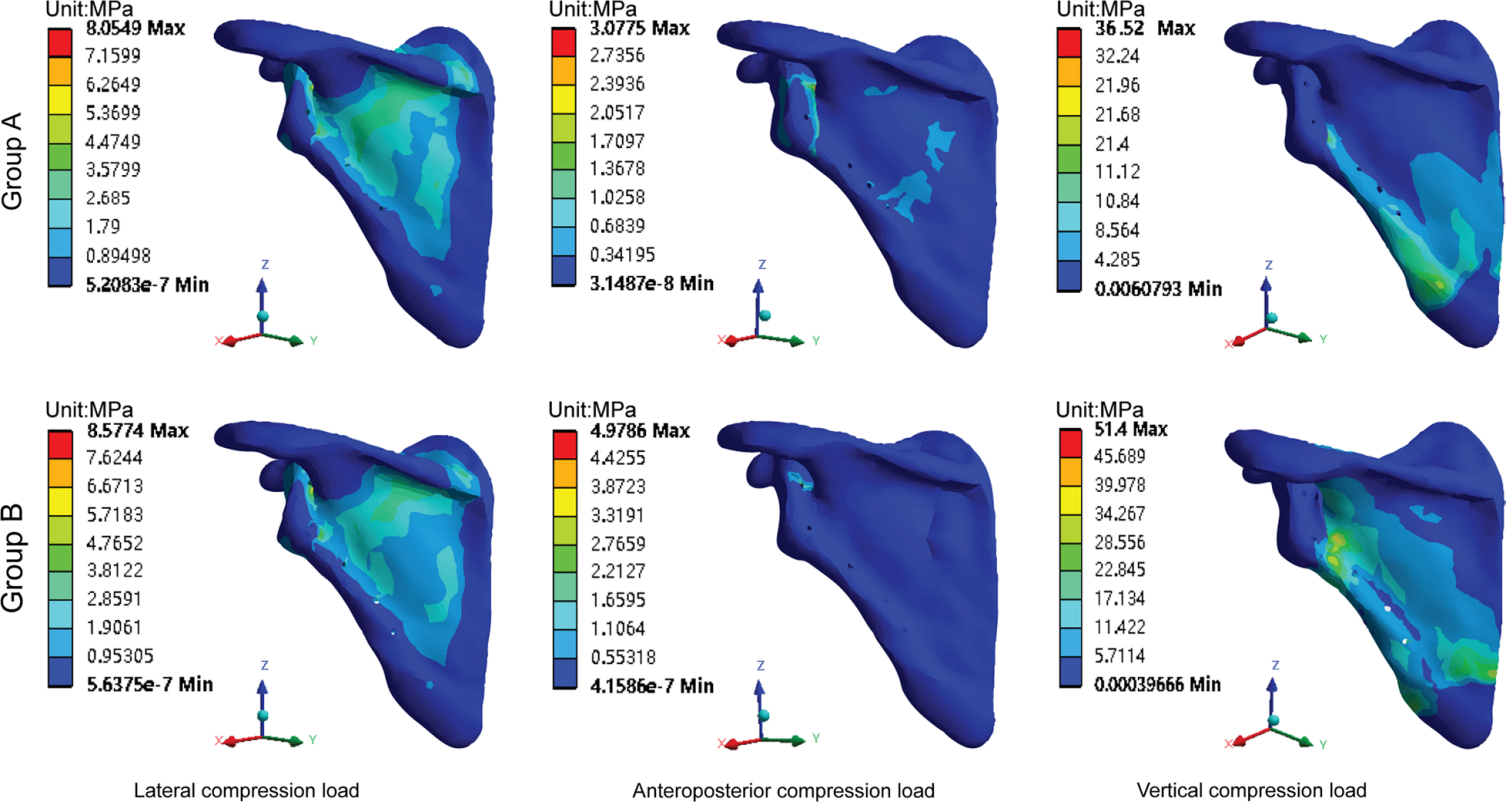
Stress distribution of the scapula. Group A (SNALCP). Group B (RP)

**Fig. 5:**
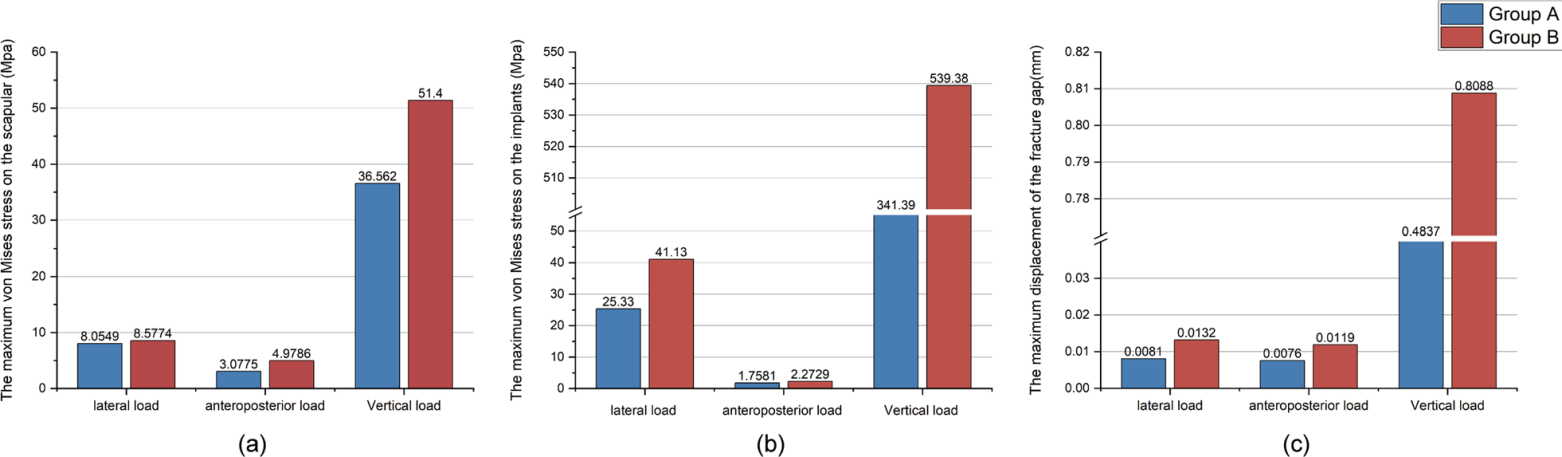
**a** Maximum von Mises stress on the scapula. **b** Maximum von Mises stress on the implants. **c** Maximum displacement of the fracture gap

The stress distribution in each group was similar for the lateral and anteroposterior compression loads. For the vertical compression load, the stress in group A was concentrated at the lateral border of the scapula, while the stress in Group B was concentrated in the scapular body (Fig. 4). The maximum stresses under the lateral, anteroposterior, and vertical compression loads on the scapula of group A (8.0549, 3.0775, and 36.5620 Mpa, respectively) were lower than those of group B (8.5774, 4.9786, and 51.4000 Mpa, respectively; Fig. 5a).

### Implant stress

**Fig. 6:**
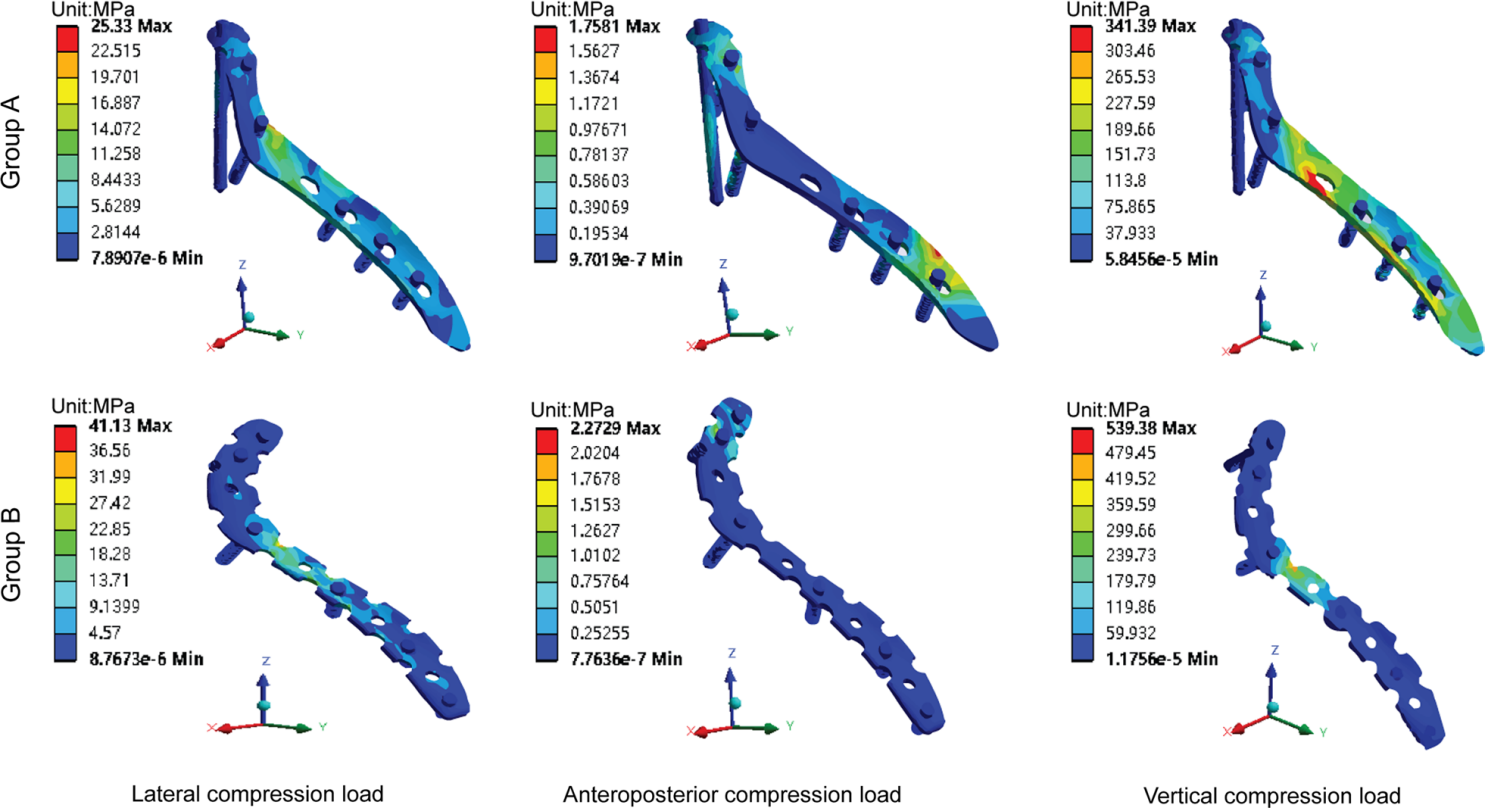
Stress distribution of the implants. Group A (SNALCP). Group B (RP)

For the lateral and vertical compression loads, the stress distribution of each group was found to be similar, mainly concentrated in the middle of the steel plates. However, the stress distribution of group A was more uniform and continuous than that of group B. For the anteroposterior compression load, the stress in group A was concentrated at the distal and proximal ends of the plate, while the stress in group B was concentrated at the proximal end of the plate and the two proximal screws (Fig. 6). Moreover, the maximum stresses under lateral, anteroposterior, and vertical compression loads on the implants of group A (25.3300, 1.7581, and 341.3900 Mpa, respectively) were lower than those of group B (41.1300, 2.2729, and 539.3800 Mpa, respectively; Fig. 5b).

### Fracture displacements

For lateral, anteroposterior, and vertical compression loads, the maximum fracture displacements of group A were 0.0081 mm, 0.0076 mm, and 0.4837 mm, respectively, which were relatively less than those of group B (0.0132, 0.0119, and 0.8088 mm, respectively; Fig. 5c).

## Discussion

Due to the high-energy nature of SNFs, most patients experience multiple traumatic injuries, such as rib fractures, rotator cuff injuries, proximal humerus fractures, and brain injuries. Moreover, SNFs are in a deep position and are often overlooked [17, 18]. In the past, most scholars have recommended conservative treatment for such fractures, but with medical advances and improved understanding of the anatomy and biomechanics of the scapular neck, orthopedic surgeons who treat SNFs agree that surgical rather than conservative treatment of unstable scapular fractures improves patient outcomes [3, 11, 12, 19, 20]. Open reduction and internal fixation in the treatment of SNFs are beneficial to early postoperative functional exercise, reduce pain, and prevent complications such as malunion, muscle imbalance, rotator cuff dysfunction, scapulothoracic dyskinesis and impingement [9, 20, 21]. However, owing to the complex anatomical structure of the scapula neck, a standard internal fixation device is lacking in clinical practice.

Jaikish et al. [9] achieved good clinical results in the treatment of displaced SNFs using reconstructive plates. They believed that RP for SNFs can ensure solid fixation and enable early functional exercise of the shoulder. However, J. Zhang et al. [12] believed that the anatomical structure of the scapula is special and that the reconstructed plate needs to be shaped repeatedly during surgery, which prolongs the surgical time and affects material strength and screw placement, resulting in an increased risk of postoperative infection, failure of internal fixation, and displacement of the fracture end. In their retrospective controlled clinical study, the authors aimed to compare the therapeutic efficacy of double-row titanium anatomical plates and reconstruction plates for extra-articular fractures of the scapula, finding that there was less intraoperative blood loss and shorter surgical duration in the anatomical plate group than in the reconstruction plate group, and that these differences were statistically significant (*P* < 0.01). However, most other fixation methods, such as Kirschner wire, hollow nail, straight locking plate, distal radius T-plate, and Y-locking plate do not conform to the anatomical shape of the scapula neck, resulting in only moderate fixation efficacy. Moreover, most fixation methods lack supporting experimental biomechanical data.

Therefore, for SNFs, we designed the SNALCP, which conforms to the anatomical characteristics of the scapular neck. For the scapular glenoid neck portion, we designed three staggered locking screws in the neck, set at a certain angle, to accomplish 3D fixation of the scapular glenoid neck and ensure more powerful internal fixation. During the operation, the plate could be attached to the bone surface of the neck and to the lateral border of the scapula with no or only slight reshaping required: this can significantly reduce the shaping time of the implant. It also effectively reduces the risk of screw insertion into the joint or damage to important tissue structures around the scapula neck (suprascapular nerves and blood vessels).

Through finite element analysis, a 3D finite element model of the SNF (Miller type IIB) was constructed that was fixed with the SNALCP and RP to compare the biomechanical characteristics of the two fixation methods. First, by comparing the maximum von Mises stresses and stress distribution on the scapula and implants of the two groups, the mechanical conduction of the two types of implants in the scapula could be evaluated [22–25]. The results of finite element analysis in the present study showed that both the SNALCP and RP effectively reduced the stress at the fracture site of the scapula under lateral, anteroposterior, and vertical loads. However, the maximum stresses of group A were smaller than those of group B. In addition, compared with group A, the stress distribution of group B has obvious high-stress concentration repercussions. In particular, when subjected to vertical compression loads, the stress in the middle of the medial border of the RP was high and highly concentrated, indicating a high risk of internal fixation failure, while the maximum stress of group A was reduced by approximately 37%. This indicates that the SNALCP has an obvious mechanical conduction advantage over RP. We speculate that this may be because the SNALCP is better fitted to the surface of the scapula, and has a larger contact area that can effectively prevent stress concentration and thereby reduce the risk of loosening of internal fixation or fracture.

The biomechanical stability of the implants in scapular fractures can be evaluated by comparing the relative displacements of the fracture gaps between the two groups of models. Overall, our data show that the maximum displacements of the fracture gaps in group A were smaller than those in group B under three different loads, indicating that the new anatomic locking plate of the scapular neck provided better stability and fixation than the reconstructed plate. We further demonstrated that the anatomical design of the scapula glenoid neck portion of the SNALCP ensures a perfect fit with the arc of the scapula glenoid neck. Three-dimensional fixation of the scapular glenoid neck was realized using three staggered locking screws, which made the screw-holding force stronger and had a better fixation effect on the scapular neck fracture. Stable fixation is essential for fracture healing to achieve better functional outcomes, particularly in patients with osteoporotic or comminuted fractures.

This study has some limitations. First, we did not take into account the influence of the muscle and ligament tissues around the scapula on the biomechanics of the scapular neck. Moreover, the shoulder joint is the most active joint in the human body, and the cyclic dynamic loads of the shoulder joint in different ranges of motion were not analyzed, which led to a difference between the data analyzed by the model and the actual data. Second, only the type IIB SNF model was analyzed, and further research is required for other classifications. In addition, there are certain differences in the scapula between different groups or sexes, and the SNALCP may not be perfect for everyone. Finally, because FEA is a simulation analysis, our results need to be confirmed by in vitro biomechanical experiments and prospective multicenter randomized controlled clinical trials.

## Conclusions

The SNALCP reduces the stress on the implant and scapula, and makes the stress more uniform and continuous, thereby ensuring better stability. This may have biomechanical advantages over RP in the treatment of SNFs. This study provides a biomechanical basis for the clinical application of SNALCP in SNFs.

## Supplementary Information

**Additional file 1.** 1.Detailed parameters of the SNALCP and the RP. 2.Detailed description of boundary and loading condition Settings.

## Data Availability

All data generated or analyzed during this study are included in the published article.

## Abbreviations

SNF: Scapular neck fracture
RP: Reconstruction plate
SNALCP: Scapular neck anatomical locking compression plate
CT: Computed tomography
DICOM: Digital imaging and communications in medicine
FEA: Finite element analysis
