# Supplementary material for: Finite element analysis of a novel anatomical locking plate for scapular neck fracture
Source: J Orthop Surg Res. 2023 Mar 31;18:262. doi: 10.1186/s13018-023-03743-3 (PMC10067195; doi:10.1186/s13018-023-03743-3)
Supplement: Supplementary file 1 — Additional file 1. 1.Detailed parameters of the SNALCP and the RP. 2.Detailed description of boundary and loading condition Settings. [file 13018_2023_3743_MOESM1_ESM.docx]

**Design of the SNALCP**

The detailed design of the SNALCP is as follows: the frontal view of the SNALCP is in the shape of a "swallow wing"; it runs from the scapular neck along the lateral border, and is divided into three parts: the scapular glenoid neck part, the junction part and the lateral border part. The scapular glenoid neck part is arched and perfectly fitted with the arc of scapular glenoid neck. A locking nail over the center of the glenoid is designed to sit at the top of the plate, and the screw hole direction is tilted 6° to the left and 21° to the bottom. Underneath, two locking screws in the direction of the coracoid process can be found. The second screw is located in the center of the glenoid neck and is angled 31° to the right. The third screw is near the junction part and is angled 16° to the right. The three staggered locking screws realize the three-dimensional fixation of the scapular glenoid neck. For the junction part, the angle is designed to match the abduction angle of the scapula (the frontal view is consistent with the abduction angle of 130°), and the overall clockwise torsion from the scapular glenoid neck part to the junction part is 5°. The lateral border part is consistent with the back of the lateral border of scapula. Four common locking joint holes are designed, and the locking method can be selected according to the need.

The SNALCP is well attached. During the operation, the plate can be attached to the bone surface of the neck and the lateral border of scapula, with no or only slight reshaping required, which can significantly reduce the shaping time of the implant.

**Parameters of the SNALCP**

The parameters of the SNALCP (7-holes) are as follows: The steel plate is 2.0mm thick and 1.0cm wide, with overall rounded corner processing. All locking holes are 3.5mm, and common holes are 3.8mm. The arched scapular glenoid neck part has a diameter of 30.0mm and a length of 35.0mm. The lateral border part is 50mm. The design has 4 locking common joint holes, and the hole spacing is 14mm.

**Parameters of the reconstruction plate**

The parameters of the reconstruction plate (10-hole) are as follows: the overall length is 124mm, the thickness is 2.0mm, the width is 1.0cm, and all the locking holes are 3.5mm in diameter (Waston Medical Instrument Co., Ltd, China).

**Boundary and loading conditions**

Three loads were applied in different directions to the two sets of models, and the corresponding boundary conditions were set (Figure 3). To simulate the most common injury mechanism of the scapular neck: a fall landing on one side, with violence transmitted through the proximal humerus to the scapular neck, a 900N lateral compression load was applied along the axis of the scapula with the scapular glenoid neck as the force surface. The medial border of the scapula was considered to be fixed (Figure 3a,b). To simulate the direct violence from the back to the chest, a 900N anteroposterior compression load was applied along the sagittal axis of human anatomy with the back side of the scapular body as the force surface. The obverse side of the scapular body was considered fixed (Figure 3c,d). To simulate a hit by a heavy object to the scapular neck from the top down, a 900N vertical compression load was applied along the longitudinal axis of the human anatomy with the acromion and the mesoscapula as the force surface. The subscapular angle was considered fixed (Figure 3e,f).
